# Thromboembolic Risk in COVID-19 Patients: Is There a Hidden Link?

**DOI:** 10.7759/cureus.18850

**Published:** 2021-10-18

**Authors:** José Cidade, Hélder Pinheiro, André Dias, Marta Santos, Bruna Nascimento, Carlos Figueiredo, Raquel Pinto, Luís Pereira, Carlos Rodrigues, Fernando Maltez

**Affiliations:** 1 Internal Medicine, Centro Hospitalar Lisboa Ocidental - Hospital Egas Moniz, Lisboa, PRT; 2 Infecciology Department, Hospital Curry Cabral, Lisboa, PRT; 3 Internal Medicine, Hospital Distrital Santarém, Santarém, PRT; 4 Pulmonology Department, Hospital Curry Cabral, Lisboa, PRT; 5 Internal Medicine, Hospital de Cascais, Lisboa, PRT; 6 Internal Medicine, Centro Hospitalar Baixo Vouga, Aveiro, PRT

**Keywords:** covid-19, sars-cov-2, pulmonary embolism, d-dimer, venous thromboembolic disease

## Abstract

Background

Although evidence has emerged indicating that patients with severe acute respiratory syndrome coronavirus 2 (SARS-CoV-2) pneumonia present a high risk of venous thromboembolism (VTE), its real incidence and best diagnosis course remain unclear. In this study, we aimed to determine the incidence of pulmonary embolism in these patients and the role of D-dimer serum level as a predictive factor of a new VTE event.

Methodology

This was a single-center retrospective observational cohort study conducted in a tertiary hospital. All patients admitted to the infectious diseases ward with SARS-CoV-2 pneumonia with clinical or laboratory criteria for suspected VTE events were eligible for inclusion in the study. The t-test or Mann-Whitney U test was used to analyze the differences between the with-VTE group and the without-VTE group.

Results

Overall, VTE incidence was registered to be 30%. Chest computed tomography angiography data revealed thrombus mainly in segmental (five patients, 71%) and subsegmental pulmonary artery branches (four patients, 57%). No thrombus on major branches was documented. D-dimer serum levels (collected at hospital admission, 48 hours before the suspected VTE event date and at suspected VTE event date) were analyzed, and, despite a consistent tendency of higher values in the with-VTE group, no statistical difference was observed. Moreover, no statistical difference was observed between the two groups in mortality rates.

Conclusions

A clear higher risk of VTE events in SARS-CoV-2 pneumonia patients was not documented, and a link between the impact of VTE occurrence and a worse prognosis was not demonstrated. Therefore, we suggest that the use of D-dimer serum level should not be used as a predictor of VTE in SARS-CoV-2 pneumonia patients.

## Introduction

Since its initial identification in December 2019, the severe acute respiratory syndrome coronavirus 2 (SARS-CoV-2) has spread worldwide and has been declared a pandemic by the World Health Organization. Although not primarily a thrombotic process, inflammation and hypoxia with acute lung injury are known mechanisms that lead to a profound inflammatory state due to cytokine production, macrophage, and endothelial activation-related processes associated with excessive inflammation and diffuse intravascular coagulation [1,2]. Therefore, initial studies have depicted these factors, along with immobilization and long hospitalization periods, as responsible for the association between high venous thromboembolism (VTE) risk and SARS-CoV-2 infection, as well as between its occurrence and poor patient prognosis [3,4].

Furthermore, SARS-CoV-2 patients usually present with markedly elevated serum levels of D-dimer with slight or no changes in partial thromboplastin time or activated partial thromboplastin time. This profile further complicates its use as an indicator of a new intercurrent VTE because elevations in D-dimer are very common in these patients; moreover, it has low specificity for VTE events [5].

In this study, we aimed to determine the incidence and risk factors of VTE in SARS-CoV-2 patients, specifically pulmonary embolism, admitted to hospital care in a medical ward. In addition, we aimed to clarify the relevance of D-dimer serum level as an indicator of a new VTE event in these patients.

## Materials and methods

Study design and population

This single-center, retrospective, observational cohort study was conducted in a tertiary hospital in Lisbon, Portugal. Data were collected from consecutive patients admitted to the infecciology hospital ward from March 1 to November 31, 2020.

Eligibility criteria included patients aged 18 or more and admitted to the infecciology hospital ward with a clinical and microbiological diagnosis of SARS-CoV-2 pneumonia. This diagnosis was made only when all the following criteria were fulfilled: (1) Confirmation of SARS-CoV-2 infection by a reverse-transcriptase polymerase chain reaction test on a nose/throat swab or a sputum sample positive for the virus; (2) clinical symptoms consistent with the diagnostic criteria of acute pneumonia (worsening respiratory insufficiency or dyspnea, cough, fever, chest pain, myalgia, vomiting, or diarrhea); (3) Radiological confirmation of lung involvement either by chest X-ray or chest computed tomography; and (4) fulfillment of Berlins’ acute respiratory distress syndrome (ARDS) criteria.

Patients were eligible for study inclusion if either a clinical (worsening of respiratory insufficiency or dyspnea, respiratory alkalosis, chest pain, syncope, or signs of deep venous thrombosis) or laboratory (variation of D-dimer serum levels of at least 10% within 48 hours) criteria of a suspected VTE event were verified during the hospital stay. These patients were submitted to chest computed tomography angiography and were further divided into two groups according to the clinical and laboratory findings: the with-VTE group or the without-VTE group. All patients with documented VTE events received anticoagulation therapy after VTE diagnosis. Thrombotic venous disease prophylaxis with low-molecular-weight heparin, as part of the treatment protocol, was administered according to the patients’ individual risk, after individual clinical evaluation, at admission.

Exclusion criteria included age less than 18 years, patients with incomplete data, and patients with a major contraindication to anticoagulation therapy prior to the diagnosis of pulmonary thromboembolism.

The Ethics Committee and Institutional Review Boards approved the study protocol as minimal-risk research, using data collected for routine clinical practice, and waived the requirement for informed consent (CES 970/2020).

Data collection

Patient demographic characteristics were recorded at baseline for all patients. For those with clinical or laboratory suspicion for a VTE event, data were recorded on comorbidities and VTE risk factors.

Clinical and laboratory data, including Wells score, anticoagulation therapy, and remdesivir and dexamethasone therapy, were also obtained for all selected patients at three time periods: at patients’ admission, 48 hours before VTE suspicion, and on the day of VTE suspicion. Patients with clinical or laboratory suspicion for a VTE event were submitted to chest computed tomography angiography, and its results and Pulmonary Embolism Severity Index (PESI) score were collected. Patients were followed until discharge or death, with hemorrhagic events (defined as any event of documented hemorrhage that motivated suspension of anticoagulation therapy) and clinical outcomes registered for further analysis.

Outcomes

The primary outcome of our study was the incidence of VTE, specifically pulmonary embolism, among patients admitted to the infecciology hospital ward with SARS-CoV-2 pneumonia with clinical or laboratory VTE suspicion. The secondary endpoints were the analysis of the influence of a VTE event on clinical outcome (discharge vs. death) and the determination of the relevance of D-dimer level as an indicator of a new VTE event.

Statistical analysis

We planned to include a total of 400 patients considering a predicted incidence rate of VTE events of 10%. Quantitative variables were expressed as medians (interquartile range) and categorical variables were expressed as numbers (percentage). The t-test or Mann-Whitney U test was used to analyze the differences between the with-VTE and without-VTE groups. Statistical analysis was conducted using SPSS software version 21.0 (IBM Corp., Armonk, NY).

## Results

**Figure 1:**
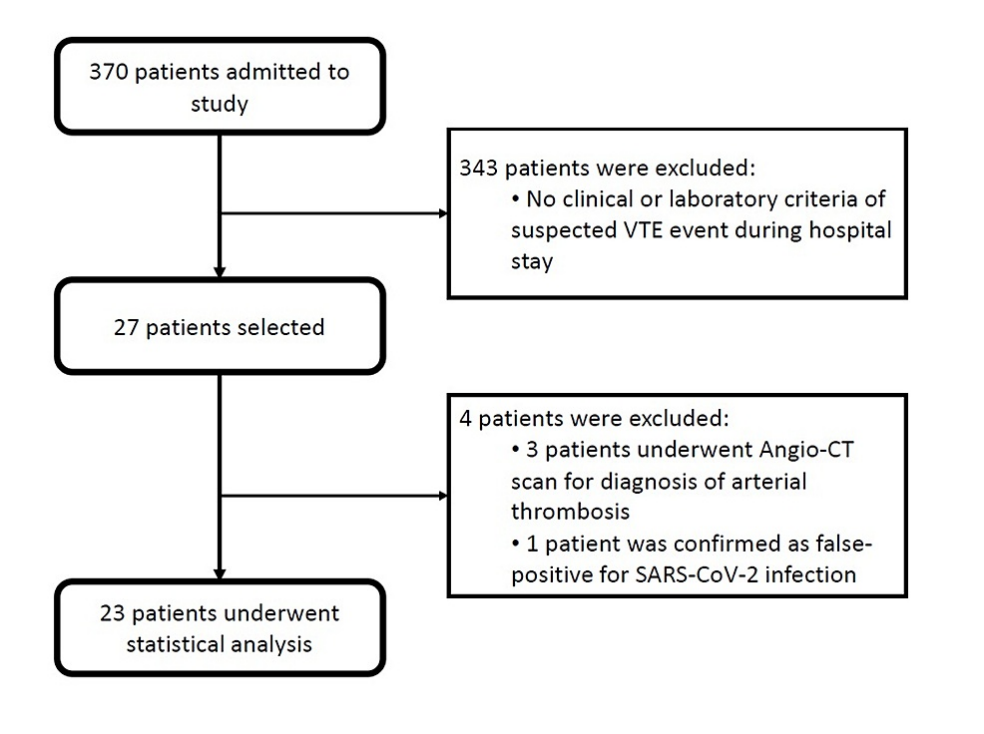
Flow diagram of the patient selection process for statistical analysis. SARS-CoV-2: severe acute respiratory syndrome coronavirus 2

In total, 370 patients were included in this study (from March 1 to November 31, 2020). Of this, 23 patients met all the inclusion criteria and underwent chest computed tomography angiography due to clinical or laboratory suspicion of a VTE event (see Figure 1).

Patient characteristics

The mean age of the study population was 67 years (range: 38-90 years) and included 13 (56.5%) males, as presented in Table 1. The majority of patients (12 patients, 52.2%) presented at least one VTE risk factor prior to hospital admission, with obesity as the most prevalent risk factor for VTE events (eight patients, 35.2%). Nine patients (39%) were also admitted to intensive care unit (ICU) care, with severe ARDS with mechanical ventilation requirement prior to suspected VTE event date, with a mean length of stay in ICU care of six days.

**Table 1:** Characteristics of the SARS-CoV-2 pneumonia patients, with suspected VTE events during hospital care (n = 23). † Prior to VTE suspicion date. SARS-CoV-2: severe acute respiratory syndrome coronavirus 2; SD: standard deviation; VTE: venous thromboembolism

Characteristic	Number (%) or mean (±SD)
Age, mean (years)	67
Range	38–90
<39	1 (4.4%)
40–49	2 (8.8%)
50–59	5 (22%)
60–69	4 (17.6%)
70–79	7 (30.8%)
80–89	3 (13.2%)
>90	1 (4.4%)
Gender	
Male	13 (56.5%)
Risk factors for VTE (prior to hospital admission)	
Major surgery (last 6 months)	1 (4.4%)
Bone fracture with prolonged immobilization (last 6 months)	0 (0%)
Active neoplasia	3 (13.2%)
Previously diagnosed thrombophilic syndrome	0 (0%)
VTE event (last 6 months)	0 (0%)
Prolonged immobilization (other causes)	1 (4.4%)
Obesity	8 (35.2%)
Active smoking habits	3 (13.2%)
Number of risk factors for VTE, per patient	
0 risk factors	11 (47.8%)
1 risk factor	11 (47.8%)
2 risk factors	1 (4.4%)
Intensive care admission†	
Number of patients	9 (39%)
Length of stay, mean (days)	6.22 (±11)

Incidence of venous thromboembolism and its predictors

VTE events were observed in seven patients with an incidence of 1.9% (7 in 370 patients) during the study. In the selected group with suspected VTE events, submitted to analysis, VTE events were observed in 30% of patients (7 in 23 patients).

The mean PESI score in patients with VTE events was 97.29 (±19.9), and chest computed tomography angiography revealed the presence of pulmonary artery thrombus mainly in segmental (five patients, 71%) and subsegmental branches (four patients, 57%). In none of the observed patients pulmonary artery thrombus on major branches was observed (Table 2).

**Table 2:** Characteristics and outcomes in the with-VTE group and without-VTE group. *p values < 0.05 for comparisons between with-VTE and without-VTE groups; ^‡ ^clinical criteria for suspected VTE (worsening of respiratory insufficiency or dyspnea, respiratory alkalosis, chest pain, or syncope). ICU: intensive care unit; DVT: deep venous thrombosis; PE: pulmonary embolism; PESI: Pulmonary Embolism Severity Index; PA: pulmonary artery; SARS-CoV-2: severe acute respiratory syndrome coronavirus 2; VTE: venous thromboembolism

Baseline characteristics	With-VTE group (n = 7)	Without-VTE group (n = 16)	P-value*
Age, mean (years)	64	68	0.56
Gender, males (n)	5 (71%)	8 (50%)	0.31
VTE risk factor: Obesity (n)	3 (43%)	5 (21%)	0.47
Previous ICU admission (n)	3 (43%)	6 (38%)	0.58
D-dimer (at hospitalar admission); ng/mL	9,376 (±1.3×10^4^)	5,891 (±1.4×10^4^)	0.85
Clinical and laboratory data (suspected VTE event date)			
Clinical criteria of suspected VTE event ‡ (n)	3 (43%)	5 (21%)	0.47
Signs of DVT (n)	0 (0%)	2 (13%)	0.45
Duration of SARS-CoV-2 pneumonia (days)	15 (±7.8)	13 (±7.4)	0.77
Mean arterial pressure (mean); mmHg	80,7 (±7.6)	84.8 (±17)	0.55
Hemoglobin, g/dL	13.9 (±2.4)	11.5 (±2.5)	0.05
Reactive protein C, mg/dL	8.5 (±9.8)	14.6 (±15.3)	0.31
D-dimer (48 hours prior to suspected VTE event date), ng/mL	8,150 (±6.4×10^3^)	2,259 (±3×10^3^)	0.09
D-dimer (at suspected VTE event date), ng/mL	13,332 (±1.3×10^4^)	11,088 (±1.6×10^4^)	0.38
Remdesivir therapy (n)	2 (29%)	6 (38%)	0.53
Dexametasone therapy (n)	2 (29%)	7 (44%)	0.42
Anticoagulation therapy (n)			0.33
Prophylactic dosage	2 (29%)	7 (44%)	
Therapeutic dosage	1 (14%)	2 (13%)	
Wells criteria for PE	1.5 (±2.1)	1.6 (±2)	0.87
Chest computed tomography angiography data			
PESI score (mean)	97.29 (±19.9)		
Thrombus on major PA branches (n)	0 (0%)		
Thrombus on segmental PA branches (n)	5 (71%)		
Thrombus on subsegmental PA branches (n)	4 (57%)		
Outcomes			
Hemorrhagic event (n)	3 (43%)	1 (6.25%)	0.07
Discharged alive (n)	7 (100%)	13 (81%)	0.31
Death (n)	0 (0%)	3 (19%)	0.32

Age, gender, and the presence of risk factors for VTE were comparable in the with-VTE and without-VTE groups, including the most prevalent of those risk factors, namely, obesity. Previous ICU admission was also not different between the two groups. Regarding clinical laboratory data, the number of patients with suspected VTE events based on clinical criteria (worsening of respiratory insufficiency or dyspnea, respiratory alkalosis, chest pain, syncope, or signs of deep venous thrombosis) and Wells criteria for pulmonary embolism score was not statistically different between the with-VTE and without-VTE groups. The mean arterial pressure at the time of suspected VTE, hemoglobin level, and C-reactive protein were also not different between the groups. To account for possible confounding factors for VTE events, the number of patients on remdesivir, dexamethasone, and anticoagulation therapies were evaluated in the with-VTE group and were not statistically different for those observed in the without-VTE group (Table 2).

**Figure 2:**
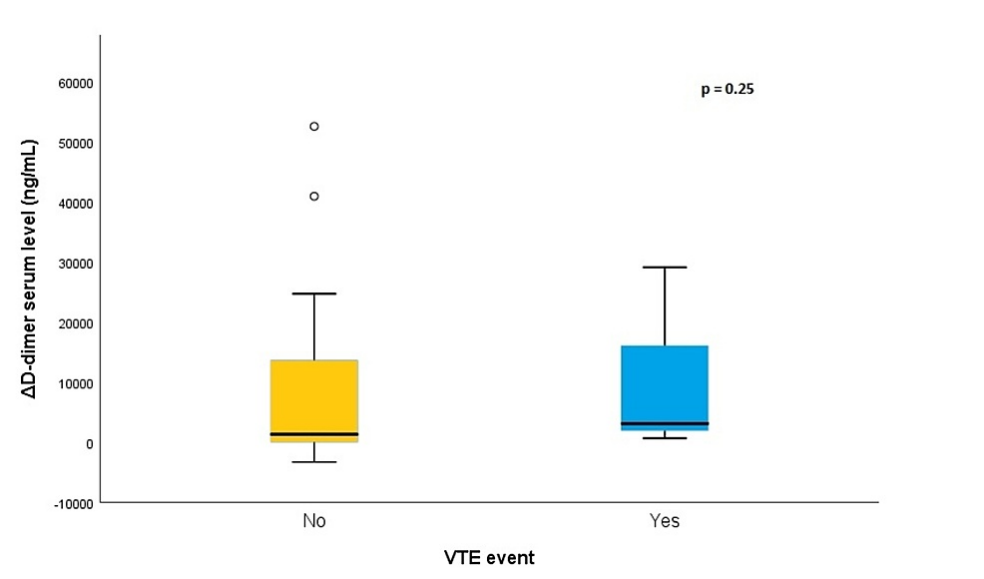
Variation of D-dimer serum level 48 hours prior to the suspected VTE event date in both with-VTE and without-VTE groups.

D-dimer serum levels at hospital admission, 48 hours prior to suspected VTE event date, and at suspected VTE event date were analyzed. Despite a consistent tendency of higher values in the with-VTE group, no statistical difference was observed. The variation of D-dimer serum level 48 hours prior to the suspected VTE event date was also comparable in the two groups (with-VTE group: mean 9,839 (± 1.1 × 10^4^) ng/mL; without-VTE group: 9,342 (±1.6 × 10^4^) ng/mL; p = 0.25) (Figure 2).

Patient outcomes

Although the number of hemorrhagic events secondary to anticoagulation therapy was not different between with-VTE and without-VTE groups, these events were clinically more severe in the with-VTE group. Two patients presented with retroperitoneal hematoma and one patient presented with limb hematoma in the with-VTE group. In the without-VTE group, one patient developed catheter-based hemorrhage due to accidental exteriorization, with no relevant registered change in D-dimer or hemoglobin serum levels after the event.

Almost all patients were discharged in both groups and no death was registered in the with-VTE group. The mortality rate of the without-VTE group was 19% but the causes of death were not clinically attributable to the occurrence of VTE or hemorrhagic complication.

## Discussion

Our study shows a reduced incidence of VTE events in SARS-CoV-2 pneumonia patients. Although this observed incidence is substantially lower than the incidence described in other recent studies [2,6,7], the increasing awareness of the association between a high VTE risk and SARS-CoV-2 infection, and the recent updates on anticoagulation recommendations in these patients, has prompted an earlier and systematic implementation of prophylactic anticoagulation therapy that can explain this lower rate [8-10]. On the other hand, as expected, the population studied versed patients admitted to the infirmary, further excluding the majority of patients admitted to ICU care with a higher risk of VTE events [11,12].

Risk factors for VTE were also observed in the studied population and no statistical association was observed with the occurrence of VTE. In the subset of patients admitted to ICU care prior to ward admission, a higher risk of VTE events was also not identified. This finding, which is not in accordance with previous evidence, may be explained by the high rate of prophylactic therapy for VTE applied in the ICU [13-16].

Further, no statistical association was found between remdesivir or dexamethasone therapies and the risk of VTE events, an issue raised in previous studies [17].

Regarding the use of D-dimer serum level as a surrogate of thromboembolic risk and a laboratory marker for suspicion of new VTE events [18], our study does not support the use of this marker as a predictor of VTE in these patients. Despite the observed tendency of higher values in the with-VTE group of D-dimer serum levels, there were no statistical differences between the two studied groups, regardless of the criteria used to raise the suspicion of a new VTE event. Furthermore, the with-VTE and the without-VTE groups remained indistinguishable even when considering the variation between values collected at admission, 48 hours before, or at the date of the suspected VTE event. Therefore, these values were not associated with new VTE events and, consequentially, were not consistent or effective in predicting VTE events. These results are consistent with evidence collected in the non-SARS-CoV-2 infection patients, considering that D-dimers are more frequently elevated in hospitalized patients and with severe infections [19]. In fact, the low positive predictive value of D-dimer has restricted its use, in most recent recommendations, to exclude new VTE events when in the normal range [20].

The occurrence of VTE events was also not associated with increased mortality. There were no deaths registered in the with-VTE group. This may be related to the fact that there was also no registered major thrombus in the chest computed tomography angiography data collected, with all patients presenting with either segmental or subsegmental branch thrombus. We hypothesize that these with the with-VTE group patients correspond, in most cases, to patients with identification of VTE events that would otherwise never be diagnosed and would not have any repercussion on their outcome. Its presence can be a reflection of higher awareness among clinicians regarding these complicating events.

Further, this is in accordance with the observed low clinical impact (considering pulmonary area affection, reduced rate of clinical suspicion criteria for VTE event, and signs of deep venous thrombosis in the analyzed population), and its low repercussion on patients’ clinical state and organ dysfunction (no unstable patients were registered at the date of suspected VTE event or posterior need of ICU care), even when superimposed with a SARS-CoV-2 infection, which usually dictates an extensive pulmonary commitment.

The impact of specific VTE events (segmental and subsegmental branch pulmonary artery thrombus) in these patients should, therefore, prompt an important clinical discussion, considering the relevance of its presence in the patients’ outcomes, and, especially, the severe hemorrhagic complications, secondary to anticoagulation therapy, observed in this subset of patients.

Our study presents some strengths, such as the systematic inclusion of patients admitted to the hospital without any selection bias, rigorous determination of laboratory D-dimer serum levels for all included patients for analysis, and continuous follow-up of all included patients until the outcome was verified. However, some limitations of our study should be acknowledged. First, it is a retrospective, single-center, small sample study. The calculated population initially idealized was not reached, reducing its statistical strength to generalize the obtained results and sustain further conclusions. Hence, the study findings need to be confirmed by larger studies. Second, in regards to VTE event characterization, venous Doppler ultrasonography of the lower limbs was not done in all patients, restricting the statistical analysis to the occurrence of pulmonary embolism and reducing the ability to identify other VTE events, such as deep vein thrombosis.

We hope that our study may contribute to improving our understanding of VTE risk in SARS-CoV-2 pneumonia patients and contribute to better hospital care for these patients.

## Conclusions

Our study has not demonstrated a clear higher risk of VTE events in SARS-CoV-2 pneumonia patients or an impact of VTE occurrence in patient prognosis. VTE events were registered in a minority of patients studied, and, in those patients, the thrombotic disease was restricted to segmental and subsegmental branches, with no cases of thrombus in major pulmonary artery branches. Furthermore, the collected data did not show any association between VTE events and increased mortality, possibly indicating a low clinical impact of VTE events.

Additionally, D-dimer serum levels, or its variation along the course of the SARS-CoV-2 infection, failed to demonstrate a good predictive accuracy of new VTE events in this specific set of patients. Therefore, this study suggests that the suspicion of a VTE event should rely on the patient’s clinical evaluation, over the sole use of D-dimer serum levels, and should be confirmed with computed tomography pulmonary angiography. Although more prospective and randomized studies are clearly necessary, this study does not support the use of this marker as a sole predictor of VTE events in the clinical diagnosis and decision-making among coronavirus disease 2019 patients.
